# Human induced pluripotent stem cell‐derived mesenchymal stromal cells regenerate diabetic ischemic muscle

**DOI:** 10.1002/btm2.70119

**Published:** 2026-02-12

**Authors:** Rohan Basu, Mackenzie K. Madison, Ali Sualeh, Theresa S. Clark, Jennifer Stashevsky, Hanaa Dakour Aridi, Nancy Zhang, Sunjay Anekal, Michael P. Murphy, Steven J. Miller, Chang‐Hyun Gil

**Affiliations:** ^1^ Department of Surgery, Division of Vascular Surgery Indiana University School of Medicine Indianapolis Indiana USA

**Keywords:** amputation, angiogenesis, chronic limb threatening ischemia, diabetes, induced pluripotent stem cells, mesenchymal stem cells, peripheral artery disease

## Abstract

Chronic limb threatening ischemia (CLTI), the most severe stage of peripheral arterial disease, affects over 500,000 patients in the United States and is associated with a 25% annual risk of amputation. Diabetic CLTI patients experience exceedingly high rates of lower extremity amputation. Many of these patients fail or are not suitable for revascularization, yet no effective non‐surgical therapies exist for this population. This study examined how human induced pluripotent stem cell (hiPSC)‐derived mesenchymal stromal cells (MSC) interrupt ischemic limb changes and stimulate muscle regeneration in a diabetic murine CLTI model. Mice treated with hiPSC‐MSC demonstrated muscle regeneration, angiogenesis, and decreased inflammation. RT‐qPCR expression of embryonic myosin heavy chain 3 (*p* < 0.01) and myoblast determination protein 1 (*p* = 0.03) mRNA was increased in ischemic muscle, at 30‐ and 7‐days post‐hiPSC‐MSC injection, respectively, indicating muscle regeneration. Vascular endothelial growth factor‐A mRNA expression was also increased at 7 days (*p* = 0.04), signifying increased angiogenic signaling. Treatment with hiPSC‐MSC decreased expression of the nicotinamide adenine dinucleotide phosphate oxidase subunit p47phox at 30 days (*p* = 0.02), suggesting decreased oxidative stress. Finally, hiPSC‐MSC‐treated mice had increased mRNA expression for the anti‐inflammatory markers, including regulatory T cell (Treg) marker Foxp3 (*p* = 0.01) at 7 days and M2‐biased macrophage marker CD206 at 7 and 30 days (*p* = 0.04 and *p* = 0.02, respectively). Our hiPSC‐MSC preparation promoted muscle regeneration, partially through Treg‐mediated M1 to M2 macrophage polarization. The use of hiPSC‐MSC to improve CLTI outcomes in diabetic patients appears promising and warrants further study.

## Graphical abstract

Diabetic CLTI patients suffer high rates of amputation and treatment failure, but cell therapy is an emerging option for “no‐option” CLTI. TALLYHO mice treated with hiPSC‐MSC showed improved limb histology, angiogenesis, and increased anti‐inflammatory markers, positioning hiPSC‐MSC as a promising option for CLTI therapy that warrants further study.

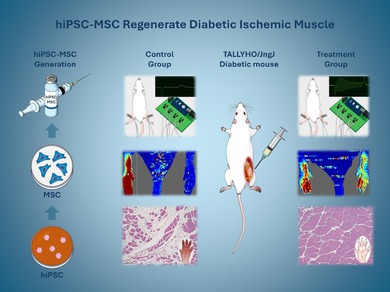

**Translational Impact Statement**

Chronic limb threatening ischemia (CLTI) substantially impacts healthcare outcomes and expenditures, with high interventional failure rates that often result in major limb amputation. Diabetic CLTI patients suffer particularly poor outcomes. This work investigated human induced pluripotent stem cell‐derived mesenchymal stromal cells as a potential scalable treatment for “no option” CLTI by utilizing polygenic‐diabetic CLTI mice, designed to model the progressive and polygenic nature of human diabetes. Cell treatments were subject to immune clearance in these immunocompetent animals, modeling the typical human use‐case.

## 1 INTRODUCTION

Chronic limb‐threatening ischemia (CLTI) is the end stage of peripheral arterial disease (PAD), affecting over 500,000 estimated patients in the United States.^1^ This condition is associated with a 25% annual risk of amputation and 60% 5‐year all‐cause mortality.^2^ CLTI patients with diabetes are at especially high risk, suffering from dramatically worse surgical limb salvage outcomes compared to nondiabetic patients.^3^ Despite these poor outcomes, there are no effective non‐surgical options to promote limb preservation in these high‐risk patients.^4^, ^5^

Cellular‐based therapy using autologous bone marrow‐derived mononuclear cells (BMD‐MNC) to treat CLTI has shown strong evidence for reducing amputation in certain subpopulations of CLTI patients.^6^, ^7^, ^8^ However, results from our laboratory^7^, ^9^ and studies from other groups^10^ have demonstrated that diabetic patients are largely refractory to autologous cell therapy.

The use of allogeneic bone marrow‐derived mesenchymal stromal cell (BMD‐MSC) preparations from young, healthy donors is a promising approach to improve regenerative therapy for CLTI patients.^11^ Unpublished clinical trial data in patients undergoing amputations for CLTI (ClinicalTrials.gov Identifier: NCT02685098) demonstrate that allogeneic MSC from a young, healthy female donor stimulates angiogenesis more effectively than autologous cells in CLTI patients, including diabetics. However, a major problem with using allogeneic BMD‐MSC for treating CLTI is the relatively low yield of cells and the resultant need to passage the cells multiple times, which can lead to phenotypic changes and replicative senescence.^12^, ^13^, ^14^, ^15^ Low cell yields also result in the need to use multiple donors to generate sufficient cells for clinical use, leading to greater variability and potential loss of efficacy of these cell preparations. Trivedi and colleagues have recently demonstrated that BMD‐MSC possess significant inter‐ and intra‐donor variability for several properties, including protein expression and immunomodulatory ability.^16^

MSC derived from human induced pluripotent stem cells (hiPSC‐MSC) provide a potentially superior source of therapeutic cells because of the ability to generate immortal cell lines, the reduced rate of phenotypic changes after multiple passages, and the relative ease of genetic modification to overexpress selected molecules to enhance function. While hiPSC‐derived MSC have been shown to counter ischemic damage in a hindlimb mouse model,^17^, ^18^ their ability to counteract effects of CLTI has not been established in the context of diabetes.

We have adapted the TALLYHO, an immunocompetent polygenic mouse model of type II diabetes,^19^, ^20^ as a CLTI model. This model of CLTI in diabetes was used to determine the ability of hiPSC‐MSC to promote tissue perfusion and skeletal muscle regeneration. Herein we show that administration of hiPSC‐MSC improves limb perfusion, enhances muscle function, and facilitates muscle regeneration in a diabetic CLTI mouse model.

## 2 MATERIALS AND METHODS

### 2.1 hiPSC culture and MSC differentiation

The cell line used in this study was derived from the umbilical cord blood of a healthy male human neonate without associated disease, obtained from Axol Bioscience (Cambridge, United Kingdom). Mycoplasma testing was performed using the American Type Culture Collection (ATCC) Universal Mycoplasma Detection Kit (ATCC, Manassas, VA, USA) to confirm the cell line was free of mycoplasma contamination before and after MSC differentiation.

Human iPSCs were maintained in mTeSR™1 complete media (Stemcell Technologies, Vancouver, BC, Canada) on Matrigel® (Corning®, Glendale, AZ, USA) in tissue culture dishes at 37°C and 5% CO_2_. Medium was changed daily, and cultures were passaged every 5–7 days.

To induce MSC differentiation from hiPSC, we used the STEMdiff™ Mesenchymal Progenitor Kit (Stemcell Technologies, Vancouver, BC, Canada) according to the manufacturer's protocol. Briefly, hiPSCs were rinsed with Dulbecco's phosphate‐buffered saline (DPBS) and then treated with 2 mL of Accutase (Gibco/Thermo Fisher Scientific, Waltham, MA, USA) for 3–5 min. Next, 8 mL of mTeSR™1 media was added. The cells were then harvested and centrifuged at 300*g* for 5 min. The cells were then seeded in a culture dish with mTeSR™1 medium, incubated for 48 h, and washed with DPBS. Subsequently, the cells were cultured in STEMdiff™‐ACF Mesenchymal Induction Medium (Stemcell Technologies, Vancouver, BC, Canada) for 4 days with daily medium changes. On Day 4, the medium was replaced with MesenCult‐ACF Plus Medium (Stemcell Technologies, Vancouver, BC, Canada), and refreshed daily for 3 days. From Days 7 to 21, the medium was changed every 3 days.

The cell preparations were then assayed to verify they met minimal criteria for multipotent MSC, as defined by the International Society for Cellular Therapy (ISCT).^21^ To verify the differentiation characteristics of MSCs, three distinct lineage differentiations were induced in vitro using the MesenCult™ Osteogenic Differentiation Kit (Stemcell Technologies, Vancouver, BC, Canada), MesenCult™‐ACF Chondrogenic Differentiation Kit (Stemcell Technologies, Vancouver, BC, Canada), and MesenCult™ Adipogenic Differentiation Kit (Stemcell Technologies, Vancouver, BC, Canada), with staining performed in accordance with each kit's protocol. Human iPSC‐MSCs at passages 4–6 were used in all experiments. MSC purity was evaluated via flow cytometry by measuring the positive expression of CD105, CD73, and CD90, and the negative expression of CD45, CD34, CD14 or CD11b, CD79a or CD19, and HLA‐DR. The differentiation efficiency was consistently high, showing more than 95% positive and less than 2% negative marker expression.

### 2.2 Animals and husbandry

All studies followed the National Research Council's Guide for the Care and Use of Laboratory Animals, were compliant with animal welfare ARRIVE guidelines, and were approved by the Indiana University School of Medicine Institutional Animal Care and Use Committee. Specific pathogen free TALLYHO/JngJ mice (male; Jackson Laboratory, Bar Harbor, ME, USA) were received at 14 weeks‐of‐age and acclimated for a minimum of 1 week prior to use. Because only male TALLYHO mice express the diabetic phenotype, female mice were not used.^19^ TALLYHO male mice develop hyperglycemia, hyperinsulinemia, hyperlipidemia, moderate obesity, and enlargement of the islets of Langerhans.^19^, ^20^ These effects are progressive in onset and better reflect the genetic status of a large percentage of the human diabetic population compared to a monogenic model homozygous for a mutation such as the *Lepr* ^*db*^ */J* (*db/db*), which is carried by very few diabetes patients. Endothelial dysfunction also has been observed in TALLYHO,^22^ which is an important characteristic of diabetes in the context of CLTI. Mice were identified by ear punch prior to surgery and housed at a maximum density of five mice per cage on sterilized contact bedding. Commercial lab rodent chow and water were supplied ad libitum and mice were housed in rooms with a 12‐h light/dark cycle at 21 ± 3°C with 30%–80% relative humidity and at least 10 changes per hour of conditioned fresh air.

### 2.3 Determination of serum glucose

Serum glucose was determined initially by analysis of serum samples from non‐fasting mice at the experiment endpoint in the Translational Core of the Indiana University Center for Diabetes and Metabolic Diseases. Subsequent measurements were made with a blood glucose meter (True Metrix Pro; Trivida Health Inc., Fort Lauderdale, FL, USA) at the time of surgery and again at the time of tissue harvest. Results were comparable between the two methods. Mice were considered diabetic if non‐fasting blood glucose was consistently ≥300 mg/dL.^23^, ^24^

### 2.4 Chronic limb threatening ischemia mouse model and hiPSC‐MSC injection

**FIGURE 1:**
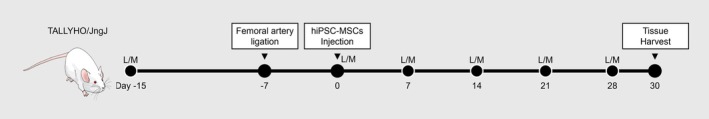
CLTI model creation. The timeline of surgery, cell delivery, LDPI, muscle testing (MT), and endpoint for the TALLYHO CLTI model is shown. The CLTI model was created through FAL 7 days prior to control or hiPSC‐MSC injection. LDPI and MT were performed weekly until harvest of tissues on post‐injection Day 30. L: Laser Doppler perfusion imaging (LDPI), M: Plantar flexion muscle testing. The diagram in the inset depicts the procedures used to derive MSC from hiPSC.

Femoral artery ligation (FAL) and excision were performed to establish severe hindlimb ischemia representative of CLTI as previously described, as this is a model of stable and severe limb ischemia.^25^ Baseline limb perfusion and plantar flexion muscle testing were determined by laser Doppler perfusion imaging (LDPI) using a Moor LD12‐HIR (Moor Instruments Inc., Wilmington, DE, USA) and an Aurora Scientific 1310A in situ muscle testing apparatus (Aurora, ON, Canada), respectively. Measurements were conducted per manufacturer guidelines. LDPI and muscle testing were performed longitudinally following model creation at set intervals as depicted in Figure 1.

On postoperative Day 7, 500,000 hiPSC‐MSC or saline vehicles were injected into the gracilis muscle by three injections of ~50 μL, not exceeding a total of 150 μL (Figure 1). Vehicle and hiPSC‐MSC‐treated mouse cohorts consisted of 5 mice per group for histological analyses and 10 mice per group for real‐time quantitative polymerase chain reaction (RT‐qPCR) analyses.

### 2.5 Tissue harvest, fixation, and histological staining

Mice were anesthetized with isoflurane inhalation. Blood was collected for serum glucose determination and preparation of serum through a closed chest needle puncture of the left cardiac ventricle. The thoracic cavity was then opened, and the vasculature was perfused with 3–5 mL phosphate buffered saline (PBS) including vascular dilator (0.1 mM adenosine and 0.01 mM sodium nitroprusside) through the left ventricle at 100–120 mmHg. Following perfusion, hindlimb muscles were rapidly dissected, carefully cut in half, and either immersion fixed in 10% neutral buffered formalin (NBF) or preserved in RNA*later* (Invitrogen/Thermo Fisher Scientific, Waltham, MA, USA) and stored at −20°C until total RNA isolation. Formalin‐fixed tissues were paraffin embedded, sectioned, and stained with hematoxylin and eosin (H&E) or Picrosirius Red (PSR) for histological or collagen analysis, respectively.

### 2.6 Immunohistochemical analyses

CD206 antigen was detected in paraffin‐embedded tissues using the VECTASTAIN® Elite ABC‐HRP Kit (Vector® Laboratories, Burlingame, CA, USA) and the Vector DAB Peroxidase (HRP) Substrate Kit with standard protocols. Antigen retrieval was performed by immersing the slides in citrate buffer (0.01 M, pH 6.0) preheated via microwave oven for 45 s, followed by heating for 3 × 5 min at 50% power, replenishing the buffer as needed between intervals. Endogenous peroxide was blocked by incubation with 3% H_2_O_2_ for 60 min at room temperature. Non‐specific protein binding was blocked with 1x protein blocking solution (Vector Animal‐free Blocker) for 60 min. The CD206/Mrc1 antibody (Abcam rabbit monoclonal, ab300621, Abcam, Waltham, MA, USA) was diluted (1:2000) with the protein blocking solution and incubated overnight at 4°C in a hydration chamber. The secondary antibody (Vector goat anti‐rabbit, biotinylated, BA1000) was diluted 1:200 in protein blocker and incubation was performed for 60 min at room temperature. The slides were briefly counterstained with Gill's 3 hematoxylin, dehydrated through graded alcohols to xylene, and coverslipped with a permanent mounting medium prior to imaging.

### 2.7 Muscle fibrosis

Relative collagen content in muscle was determined by thresholding using ImageJ to determine the area of PSR staining as previously described.^26^ Threshold analysis was performed using the green channel of the RGB stack, and a grayscale value of 90 was used to standardize values for PSR stained areas between samples.

### 2.8 Capillary density

Formalin‐fixed paraffin‐embedded muscle sections were incubated with Trilogy™ pretreatment solution (Cell Marque™ Tissue Diagnostics, Rocklin, CA, USA) for deparaffinization and antigen retrieval, washed with tris‐buffered saline with Tween 20 (TBST), and blocked with 1× Animal‐Free blocker (Vector Laboratories, Newark, CA, USA). Sections were incubated with a 1:50 dilution of the endothelial‐specific Griffonia Simplicifolia lectin I (GSL I) isolectin‐B4 (Vector Laboratories) for 60 min, followed by washing and a second incubation with a 1:500 dilution of wheat germ agglutinin (WGA)‐555 lectin (Invitrogen/Thermo Fisher Scientific) to label muscle fibers. Nuclei were detected by a 5‐min incubation with a 1:500 dilution of DAPI (Thermo Fisher Scientific). Tissues were coverslipped with Vector Express (Vector Laboratories) as per the manufacturer's protocol. Fluorescent images were obtained for 3–5 representative sections from 2 to 3 nonsequential serial sections per mouse muscle. Capillaries were counted in a field of view and normalized to unit area.

### 2.9 Imaging

Digital images of muscle sections were acquired with a Leica DM 5000B microscope, a DMC 6200 digital camera, and LASX imaging software (Leica Microsystems, Inc., Buffalo Grove, IL, USA). Multiple non‐sequential serial tissue sections from each mouse muscle were evaluated for histopathological characteristics. Morphometric measurements were made from stored images with ImageJ.^27^

### 2.10 RT‐qPCR

Relative differences in muscle mRNA expression for various molecules were determined using reverse transcription real‐time quantitative PCR (RT‐qPCR) as previously described in detail.^28^ Briefly, tissues were perfused with cold PBS followed by RNA*later* (Invitrogen/Thermo Fisher Scientific) and total RNA was isolated using the RNeasy Fibrous Tissue Mini Kit (Qiagen, Valencia, CA, USA), which included on‐column DNase digestion. RNA concentration and quality were determined using a NanoDrop ND1000 Spectrophotometer (NanoDrop Products, Wilmington, DE, USA). Aliquots of purified total RNA (1.0 μg) were enzymatically treated to remove potential contaminating genomic DNA (DNA free™, Invitrogen) and then reverse transcribed using Ready‐To‐Go You‐Prime First‐Strand Beads (GE Healthcare/Amersham Biosciences, Piscataway, NJ, USA) with random decamer priming. For RT‐qPCR, aliquots of cDNA (5.0 μL, 1: 25 dilution) were combined with murine‐specific primers and probes for MyH3, MyoD1, VEGF‐A, Mrc1, IL‐10, p47phox, FoxP3, or hypoxanthine‐guanine phosphoribosyltransferase (HPRT) endogenous control (TaqMan™ Gene Expression Assays, Thermo Fisher/Applied Biosystems) in the presence of a PCR master mix (TaqMan™ Fast Advanced Master Mix, Thermo Fisher/Applied Biosystems). Reactions were run in triplicate on an ABI 7500 Real‐Time PCR System (Applied Biosystems, CA, USA) using relative quantification (ddCt) with standard two‐step cycling conditions (40 cycles). Differences in PCR product yields between groups were determined by comparing the fold differences (RQ) between target mRNA after normalization to HPRT.

## 3 RESULTS

### 3.1 Differentiation of hiPSC‐MSC

We successfully induced the differentiation of hiPSCs into MSCs, as confirmed by specific staining for adipocytes, chondrocytes, and osteocytes. Flow cytometry analysis demonstrated that the differentiated cells were positive for expression of CD105, CD73, and CD90, and negative for expression of CD45, CD34, CD19, and HLA‐DR (Figure S1). According to these results, the hiPSC‐MSCs satisfy the criteria established by the International Society for Cellular Therapy.^21^ The cells were also confirmed to be free of mycoplasma contamination before and after differentiation into MSCs (Figure S2).

### 3.2 Determination of diabetic phenotype

Serum glucose concentrations were measured in non‐fasting mice. Mean values were similar in both vehicle and cell‐treated groups (570.5 ± 11.6 and 539.9 ± 21.9 mg/dL, respectively), confirming a diabetic phenotype and demonstrating hiPSC‐MSC administration did not affect serum glucose levels (Figure S3). Because the diabetic phenotype is not fully penetrant in the male TALLYHO mice, blood glucose measurements were made at the time of CLTI model creation as well as at tissue harvest. The prevalence of the non‐diabetic phenotype (≤300 mg/dL) was approximately 10% of the total mice number.

### 3.3 hiPSC‐MSC improve hindlimb muscle perfusion and function

**FIGURE 2:**
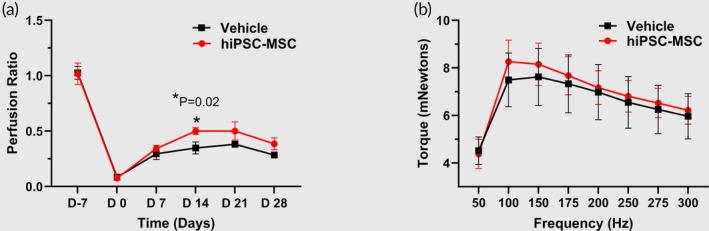
hiPSC‐MSC increased muscle perfusion and function. (a) TALLYHO hindlimb blood perfusion ratio (ligated: control) significantly increased in hiPSC‐MSC‐treated mice 14 days post‐injection and remained greater than vehicle control values out to 28 days post‐FAL. Significance was determined using one‐tailed *t*‐tests. (b) Plantar flexion muscle peak torque was greater in hiPSC‐MSC‐treated ischemic muscle compared to vehicle‐treated ischemic muscle at 14 days post‐cell injection (*n* = 5/group).

Tissue perfusion was determined by LDPI at baseline prior to surgery, 1 day following surgery, and then at 7‐day intervals up to the time of euthanasia (Figure 2a). Injection of hiPSC‐MSC was performed 7 days after model creation to establish preexisting ischemic muscle damage before cell injection. This method duplicates the intended clinical use of cells in patients with preexisting ischemic damage and muscle wasting. The ratio of perfusion in the ischemic limb to the contralateral control (non‐ligated) limb was significantly greater than vehicle controls at 7 days following hiPSC‐MSC injection (hiPSC‐MSC = 0.50 ± 0.03 vs. Veh = 0.35 ± 0.05, *p* = 0.02, Figure 2a). This ratio remained above control values until tissue collection, though it was not statistically significant, suggesting the most substantial perfusion effects from cell treatment occur shortly after injection and are sustained for 4 weeks (Figure 2a and Figure S4). A parallel analysis of plantar flexion muscle function indicated that administration of hiPSC‐MSC increased torque at 14 days post‐cell injection at higher stimulation frequencies, although this did not reach statistical significance at any time point (Figure 2b). No difference in muscle fatigue between groups was detected at any time point following cell injection (data not shown), suggesting that, while hiPSC‐MSC treatment aids muscular mass regeneration, the effects on muscular function are more subtle. LDPI and plantar flexion assays can also demonstrate variability that may underestimate true effect sizes of smaller magnitude changes.

### 3.4 hiPSC‐MSC improve muscle histology

**FIGURE 3:**
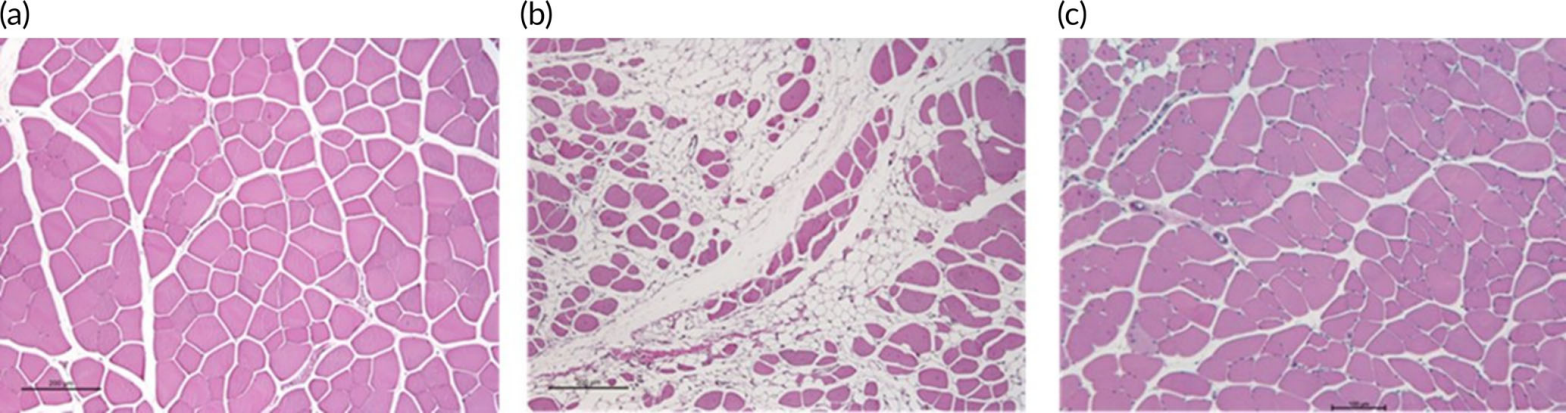
hiPSC‐MSC stimulate muscle regeneration. Representative images of H&E‐stained gastrocnemius muscle from non‐ischemic (a), vehicle‐treated ischemic (b), and hiPSC‐MSC‐treated groups (c). Administration of hiPSC‐MSC post‐ischemia normalized the ischemia‐induced muscle fiber loss and increased adiposity to near non‐ischemic levels (magnification = ×100).

Gastrocnemius muscle sections were evaluated for characteristics of ischemic damage including presence of polymorphic muscle fibers, necrotic muscle fibers, small regenerating muscle fibers, regenerated muscle fibers, intramuscular adipocytes, intramuscular macrophages, and collagen deposition. Non‐ischemic control tissue showed normal histology (Figure 3a), whereas vehicle‐treated ischemic muscle exhibited monomorphic myocytes with centralized nuclei, macrophages, cellular infiltrates, necrotic muscle fibers, and adipocytes (Figure 3b). The hiPSC‐MSC‐treated ischemic muscle had isolated areas of muscle fibers with centralized nuclei, suggesting muscle regeneration (Figure 3c). The hiPSC‐MSC treated muscle showed significantly reduced infiltrates and adipocytes compared to ischemic gastrocnemius muscle (Figure 3c). Similar results were seen with control, ischemic, and hiPSC‐MSC‐treated tibialis muscle (results not shown).

H&E‐stained gastrocnemius sections were independently scored by two experts across five domains as specified in Table S1. Sections were scored based on percentages of the section with the specified pathology, with 0 indicating no change, 1 indicating <25%, 2 indicating 26%–50%, 3 indicating 51%–75%, and 4 indicating 76%–100%. Mouse mean values were analyzed due to high variability with analysis by sections. The hiPSC‐MSC‐treated group demonstrated a similar degree of cellular infiltrates when compared with the non‐ischemic control group. The hiPSC‐MSC‐treated group also had significantly less fatty deposition than the vehicle‐treated group, with a trend to decreased degrees of pathology in the other four groups (Table S1).

**FIGURE 4:**
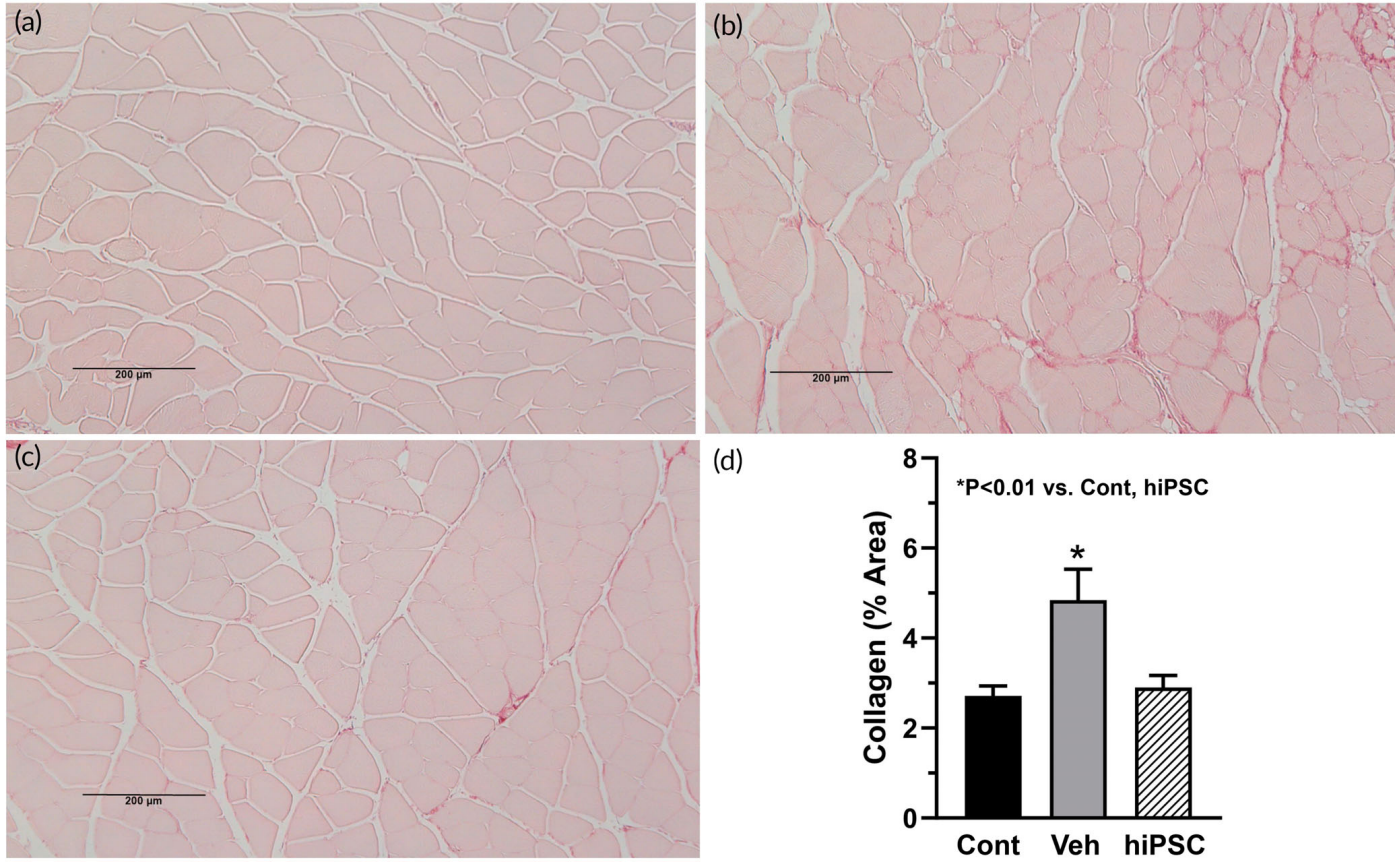
hiPSC‐MSC prevent muscle fibrosis. (a–c) Representative images of PSR‐stained gastrocnemius muscle (magnification = ×100) from non‐ischemic control, ischemic control, and ischemic hiPSC‐MSC‐treated mice, respectively. (d) Fibrosis was quantified by threshold analysis with ImageJ, and results indicated increased collagen deposition in the ischemic vehicle‐treated gastrocnemius muscle (Veh) compared to the non‐ischemic control limb (Cont). hiPSC‐MSC treatment (hiPSC) muscle fibrosis was comparable to non‐ischemic control levels (*n* = 4–5/group; significance was determined by two‐tailed *t*‐test).

Picrosirius red (PSR) staining of gastrocnemius muscle tissues was used to determine changes in collagen content (Figure 4). Collagen content was expressed as a percentage of total tissue area with visible PSR stain. Collagen was increased in vehicle‐treated ischemic hindlimb muscle 30 days post‐model creation (4.84% ± 0.69%, Figure 4b) when compared to non‐ischemic control muscle (2.72% ± 0.22%, *p* < 0.01, Figure 4a). However, collagen content in hiPSC‐MSC‐treated ischemic muscle (2.90 ± 0.27%, Figure 4c) did not differ from control levels (Figure 4d). A higher magnification image is displayed in Figure S5, demonstrating low background staining and high intercellular PSR staining in tissue sections with more fibrosis.

### 3.5 hiPSC‐MSC‐stimulate angiogenesis

**FIGURE 5:**
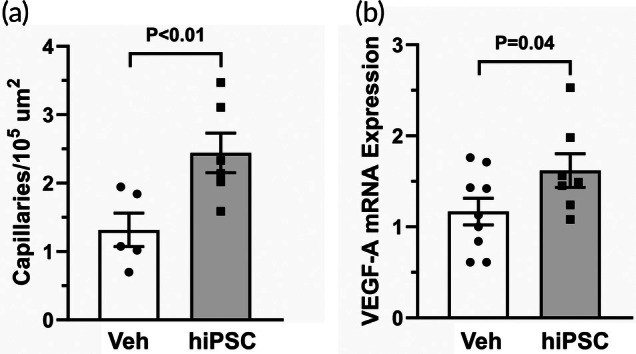
hiPSC‐MSC stimulated angiogenesis in ischemic gastrocnemius. (a) The number of capillaries per unit area was determined as an index of muscle angiogenesis in gastrocnemius from vehicle and hiPSC‐MSC‐treated mice. (b) VEGF‐A mRNA expression was determined by RT‐qPCR at 7 days post‐cell injection and the result is expressed as relative fold change after normalization to HPRT. Veh = vehicle‐treated ischemic muscle, hiPSC = hiPSC‐MSC‐treated ischemic muscle. Significance was determined using one‐tailed *t*‐tests.

To determine if administration of hiPSC‐MSC to ischemic muscle stimulated angiogenesis, capillary density in GSL I IB4‐lectin‐stained gastrocnemius muscle was measured. The results indicated that a significant increase in capillary density was present 30 days after injection in the hiPSC‐MSC‐treated group (2.44 ± 0.29) compared to the control group (1.32 ± 0.25, *p* < 0.01, Figure 5a). Because an increase in capillary density/angiogenesis had been observed following administration of hiPSC‐MSC (Figure 5a), expression of vascular endothelial growth factor A (VEGF‐A), a known angiogenic growth factor, was measured using RT‐qPCR. VEGF‐A mRNA expression was increased in gastrocnemius 7 days post cell injection (hiPSC = 1.62 ± 0.19 vs. Veh = 1.17 ± 0.15, *p* = 0.04, Figure 5b), consistent with early angiogenesis after hiPSC‐MSC treatment, further demonstrated by the increased capillary density observed at 3 days.

### 3.6 hiPSC‐MSC‐induced changes in expression of messenger RNA related to muscle regeneration and inflammation

**FIGURE 6:**
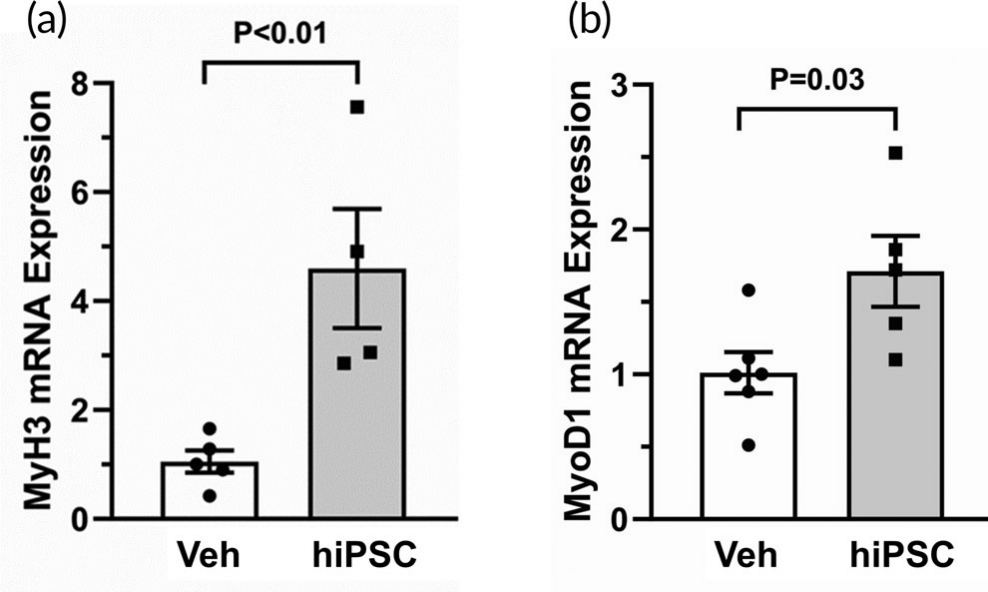
RT‐qPCR analysis of muscle regeneration markers. Relative fold changes in expression were determined by RT‐qPCR for (a) embryonic myosin heavy chain (MyH3) and (b) myoblast determination protein 1 (MyoD1) mRNA in ischemic gastrocnemius muscle 30 and 7 days after hiPSC‐MSC injection, respectively. Veh = vehicle‐treated ischemic muscle, hiPSC = hiPSC‐MSC‐treated ischemic muscle. Significance was determined using two‐tailed *t*‐tests.

Molecular markers associated with muscle regeneration, including embryonic myosin heavy chain (MyH3) and myoblast determination protein 1 (MyoD1), were assessed. Assessment of mRNA expression using RT‐qPCR showed that both markers were significantly increased in gastrocnemius muscle treated with hiPSC‐MSC compared to vehicle‐treated control muscle following cell injection (MyH3 hiPSC = 4.60 ± 1.09 vs. Veh = 1.05 ± 0.20, *p* < 0.01, Figure 6a) (MyoD1 hiPSC = 1.65 ± 0.21 vs. Veh = 1.01 ± 0.14, *p* = 0.03, Figure 6b). Additionally, MyH3 expression in tibialis muscle 30 days following administration of hiPSC‐MSC was 6.6 ± 2.1 (*n* = 7) hiPSC‐MSC‐treated versus 2.4 ± 0.7 (*n* = 4) vehicle‐treated (*p* = 0.03, data not shown).

**FIGURE 7:**
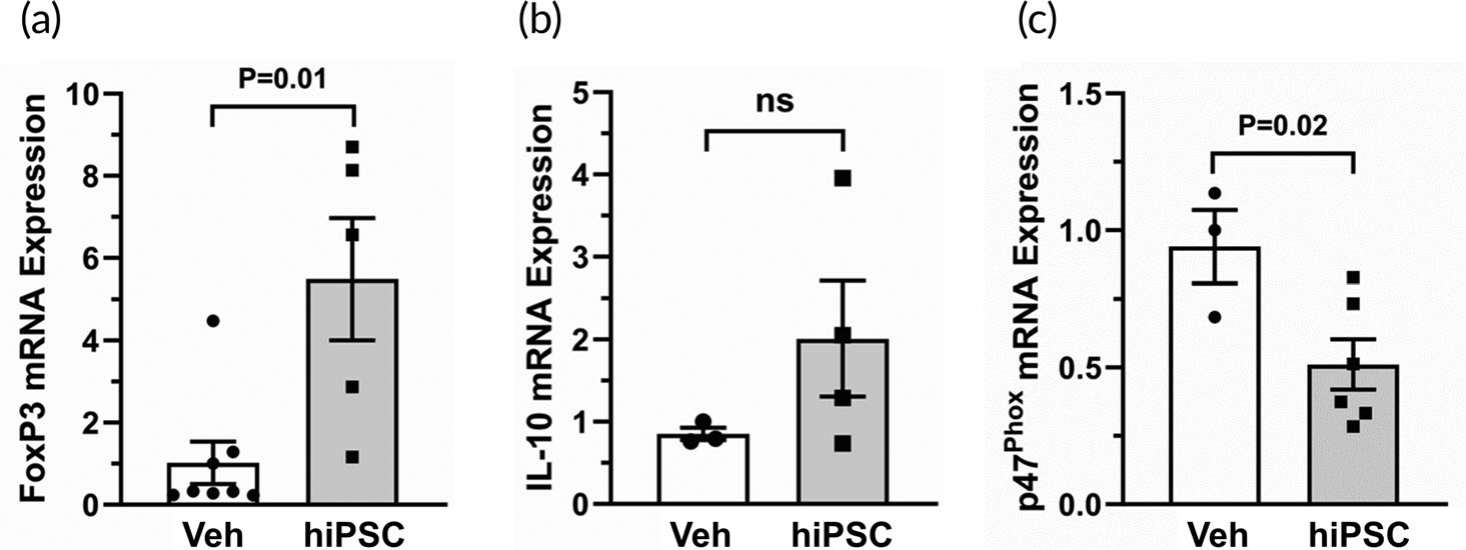
PCR analysis of markers related to Treg function and oxidant stress. (a) Relative fold changes in expression for murine FoxP3 in ischemic gastrocnemius muscle were determined at 7 days after cell injection. (b, c) IL‐10 and p47^phox^ expression were determined 30 days following cell injection. Veh = vehicle‐treated ischemic muscle, hiPSC = hiPSC‐MSC‐treated ischemic muscle. Significance was determined using two‐tailed *t*‐tests.

Molecular markers related to regulatory T cell (Treg) presence and function were examined, including forkhead box P3 protein (FoxP3) and interleukin 10 (IL‐10). Both markers were elevated in hiPSC‐MSC gastrocnemius muscle compared to controls 3 days after cell injection (FoxP3 hiPSC = 5.49 ± 1.49 vs. Veh = 1.02 ± 0.51, *p* = 0.01, Figure 7a) (IL‐10 hiPSC = 2.01 ± 0.70 vs. Veh = 0.85 ± 0.08, *p* > 0.05, Figure 7b), though the increase in IL‐10 was not significant, likely due to low expression in the vehicle‐treated group and high variability in the hiPSC‐MSC‐treated group.

Because MSC have been shown to have general anti‐inflammatory properties^29^ expression of the pro‐oxidant/pro‐inflammatory NADPH oxidase subunit p47^phox^ was determined. Injection of hiPSC‐MSC resulted in a significant decrease in p47^phox^ mRNA (0.51 ± 0.09) versus control‐treated gastrocnemius after 3 days (1.17 ± 0.40, *p* = 0.02, Figure 7c). This result is consistent with the observed increase in the anti‐inflammatory cytokine IL‐10.

**FIGURE 8:**
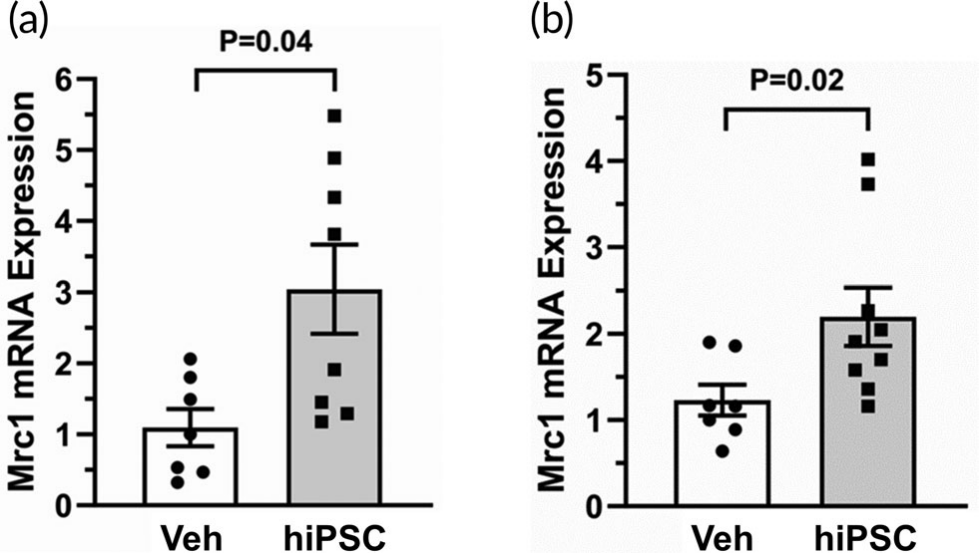
hiPSC‐MSC stimulate regenerative macrophages. M2‐biased macrophage marker Mrc1 (CD206) mRNA expression changes were determined by RT‐qPCR at (a) 7 days and (b) 30 days following injection of hiPSC‐MSC. Results are expressed as relative fold change after normalization to HPRT. Veh = vehicle‐treated ischemic muscle, hiPSC = hiPSC‐MSC‐treated ischemic muscle. Significance was determined using one‐tailed *t*‐tests.

Because muscle regeneration is highly dependent on the presence of the M2‐biased macrophage phenotype^30^ mRNA expression of the mannose receptor C‐Type 1 (Mrc1/CD206), a marker of M2‐biased macrophages,^31^ was determined. Injection of hiPSC‐MSC increased Mrc1 mRNA expression at both 7 (hiPSC = 3.04 ± 0.63 vs. Veh = 1.10 ± 0.26, *p* = 0.04, Figure 8a) and 30 (hiPSC = 2.20 ± 0.34 vs. Veh = 1.23 ± 0.18, *p* = 0.02, Figure 8b) days after hiPSC‐MSC treatment as determined by RT‐qPCR analysis. The presence of Mrc1/CD206‐positive cells was confirmed using immunohistochemistry (Figure S6). The mean density of CD206‐positive cells was higher in the hiPSC‐MSC group (hiPSC = 30.09 ± 22.57 cells/mm^2^ vs. Veh = 7.81 ± 22.57 cells/mm^2^).

## 4 DISCUSSION

The goal of this study was to understand the effect and mechanisms of hiPSC‐MSC‐mediated mitigation of ischemic damage to hindlimb muscle. A diabetic CLTI model was chosen, as human diabetic patients are a particularly challenging subpopulation to treat.^3^ Many studies have been performed using cells to treat ischemic hindlimbs in murine models.^17^, ^18^, ^32^, ^33^, ^34^, ^35^, ^36^ However, only one study to date has examined diabetic animals in this context and it was performed in leptin knockout mice, which do not reflect the majority phenotype of diabetic people.^37^ To the authors' knowledge there are no other publications demonstrating successful treatment of hindlimb ischemia using cell therapy in a polygenic diabetic murine model, such as TALLYHO.

While there are no studies directly comparing TALLYHO to other mouse strains used for models of cardiovascular diseases, several other mouse strains are commonly used. Pathologically, TALLYHO mice exhibit impaired endothelium‐dependent vascular relaxation, increased superoxide generation, and enhanced contractile responses in the aorta, with critical roles for prostaglandin H2/thromboxane A2 receptors and cytochrome P450 products.^38^ In CLTI models, these polygenic diabetic mice demonstrate muscular fibrosis and adipose deposition.^39^ BALB/c mice, commonly used as a model for PAD, develop pronounced muscle atrophy, fibrosis, adipose accumulation, and poor perfusion recovery after limb ischemia, making them a common model for CLTI.^40^, ^41^ A common monogenic diabetic mouse model, the Lepr(db/db) mouse, demonstrates impaired neovascularization, increased vascular inflammation and remodeling, and severe endothelial dysfunction.^42^, ^43^ While there are some differences between strains in the pathological response to hindlimb ischemia, MSC appear to aid angiogenesis and inflammation across various strains.^17^, ^44^, ^45^ Diabetic mouse strains, however, appear to commonly demonstrate endothelial dysfunction, which is a key characteristic in the relationship between diabetes and CLTI.^46^

A distinguishing feature of this study is the use of immunocompetent TALLYHO mice. Most cell therapy research is conducted using immunocompromised mouse strains, such as Nod‐SCID or BALB/c.^33^, ^34^, ^35^, ^36^ While an immunocompromised state allows for longer survival of xenogeneic and allogeneic cells, use of immunocompetent mice more appropriately mirrors the proposed therapeutic approach in humans, with allogeneic MSC.^47^ Accordingly, there is a limitation to cell viability in immunocompetent subjects, as most cells only last a few days.^48^ Previous work from our laboratory investigating the effect of human MSC administration on IL‐10 levels in an immunocompetent mouse model demonstrated a transient increase in human IL‐10 levels followed by a progressive increase in mouse IL‐10 for the next 21 days.^49^ Paracrine effects of MSC are well‐established.^50^, ^51^ Our results are consistent with the idea that, despite their brief lifespan in immunocompetent subjects, MSC can exhibit a potent paracrine effect that influences native cell activity.

Significant variation exists for cell treatment in ischemic hindlimb models. The decision for injection of MSC into the gastrocnemius muscle is based on previous unpublished observations from our group that demonstrate equivalent results with injection into the gracilis and gastrocnemius muscles. Additionally, pre‐clinical data demonstrate minimal additional effects for escalating doses for MSC in ischemic pathology, as well as a wide range of MSC doses, with most studies injecting either 500,000 or 1 million cells per treatment.^52^ Previous unpublished work from our laboratory also demonstrated no difference in outcomes following injection with 500,000 cells (low dose) or 1 million cells (high dose).

Most studies to date using cells to treat ischemic hindlimbs created through femoral artery ligation/excision use one timepoint for both model creation (ligation) and treatment (cell injection), which leaves no window for ischemic damage to occur. In contrast, our model induces ischemia with cell treatment occurring a week after onset of ischemia. The rationale behind this delay is to avoid the inflammatory cascade that is activated as a response to ischemic insult. Thus, this experimental design avoids any confounding increase in circulating cytokines due to the innate ischemic response by waiting until after the resolution of this response. This approach ensures that molecular changes are due primarily to cell treatment alone. Additionally, delaying administration of cell treatment better parallels clinical treatment paradigms, under which patients are treated after they present with symptoms or evidence of existing ischemic muscular injury.

In the setting of inflammation and ischemic muscle damage, macrophages undergo phenotypic switching, during which macrophages transition between the M1‐biased (pro‐inflammatory) and the M2‐biased (anti‐inflammatory) phenotypes.^45^, ^53^, ^54^ The M2 phenotype is known to be involved in muscle regeneration through regulatory T cell control.^30^ Our data is consistent with this principle demonstrating exogenous hiPSC‐MSC treatment promotes muscle regeneration through IL‐10/FoxP3‐mediated M1‐M2 phenotypic switch, evidenced by increased expression of M2‐macrophage markers on RT‐qPCR and increased M2‐macrophage staining on immunohistochemistry. This notion is further confirmed by decreased expression of downstream markers of oxidative stress and inflammation such as p47^phox^. However, these trends were not consistent for each marker at every timepoint, likely reflecting the dynamic nature of tissue repair. In particular, the increased IL‐10 and M2‐macrophage markers at later timepoints are consistent with prior studies using MSC in CLTI models, demonstrating a continued anti‐inflammatory effect of cell therapy that parallels tissue repair and reperfusion.^45^, ^55^, ^56^ The increased expression of Foxp3 at the 7‐day timepoint is consistent with the increased recruitment of Treg cells during the subacute phase of inflammation, as inflammation subsides and tissue repair progresses.^57^ In addition to Treg‐mediated pathways, muscle regeneration was further confirmed by increased expression of independent markers of muscle regeneration, MyH3 and MyoD1, with early angiogenic response demonstrated by increased capillary density and expression of VEGF.

While there are many strengths to this study, some limitations exist. The lack of gender representation and genetic control may limit translatability to human subjects. Variability observed in expression of certain markers may be due to natural animal variability or variability in delivering hiPSC‐MSC into the muscle. Paracrine effects of exogenous human MSC also may not parallel murine cells due to differences in cytokine structure and receptor function between species, which could mute the potential effects of human MSC compared to murine MSC. An example of one such difference is in the immunosuppressive role of MSC‐mediated indoleamine 2,3‐dioxygenase in humans, a pathway that is instead modulated by inducible nitric oxide synthase in mice.^58^

Future work by our group will include alginate hydrogel encapsulation of MSC, with the goal of amplifying the paracrine effects of cells by protecting them from immune‐mediated clearance or destruction and altering the paracrine secretome.^59^ Additional studies to alter the phenotype of MSC to produce more anti‐inflammatory and regenerative cytokines are also planned, including pretreatment at low oxygen tension and high glucose concentrations to mimic microenvironmental conditions in ischemic diabetic muscle.^60^, ^61^, ^62^ Ongoing investigations will also aim to further elucidate the comparative efficacy of different cell types, including vertebral body adherent (vBA) MSC.^39^ Once the parameters of cell treatment, including cell type, delivery method, dose, and formulation, have been optimized, transcriptomic analyses are planned, using RNA sequencing analyses on muscle from the TALLYHO ischemic hindlimb model treated with various MSC types.

## 5 CONCLUSIONS

The current study was performed to assess the ability of hiPSC‐MSC to ameliorate damage from limb ischemia, like that associated with CLTI, in a polygenic diabetic mouse model that better models human type 2 diabetes. Taken together, the results indicate hiPSC‐MSC promote an environment that mitigates ischemic muscle damage, reduces inflammation, and improves muscle function by supporting increases in perfusion, muscle regeneration, and angiogenesis. The effectiveness of MSC derived from hiPSC, coupled with their abundance and widespread availability, makes hiPSC‐MSC a more practical formulation for cell therapy in CLTI patients.

## AUTHOR CONTRIBUTIONS

**Rohan Basu:** Conceptualization; investigation; writing—original draft; methodology; visualization; writing—review and editing; formal analysis; data curation; software; validation. **Mackenzie K. Madison:** conceptualization; investigation; writing—original draft; writing—review and editing; methodology; formal analysis; data curation. **Ali Sualeh:** Investigation. **Theresa S. Clark:** Conceptualization; investigation; methodology; software. **Jennifer Stashevsky:** Investigation; visualization. **Hanaa Dakour Aridi:** Investigation; writing—review and editing. **Nancy Zhang:** Investigation. **Sunjay Anekal:** Investigation. **Michael P. Murphy:** Conceptualization; funding acquisition; supervision; writing—review and editing; visualization; formal analysis; methodology; project administration; resources. **Steven J. Miller:** Supervision; formal analysis; writing—review and editing; visualization; methodology; conceptualization; investigation; writing—original draft; data curation. **Chang‐Hyun Gil:** Writing—original draft; supervision; conceptualization; investigation; methodology; visualization; writing—review and editing; formal analysis; data curation; software; validation.

## FUNDING INFORMATION

This work was supported by the Cryptic Masons Medical Research Foundation, Brownsburg, IN.

## CONFLICT OF INTEREST STATEMENT

The authors declare no conflict of interest.

## Supporting information


**Figure S1.** Analysis of hiPSC‐MSC. (A, B) Morphology of hiPSC‐MSC. (C) Surface marker expression of hiPSC‐MSC as determined by flow cytometry. (D) Microscopic images of hiPSC‐MSC derived adiopocytes, osteoblasts, chondroblasts.
**Figure S2.** Gel electrophoresis of PCR confirmation of mycoplasma‐free cell line. The left panel demonstrates no mycoplasma DNA in the hiPSC cell preparation prior to differentiation. The right panel demonstrates no mycoplasma DNA in the hiPSC cell preparation after MSC differentiation.
**Figure S3.** hiPSC‐MSC administration did not affect serum glucose levels in diabetic phenotype. Average serum glucose concentrations, as determined via a glucometer, in the control and hiPSC‐MSC‐treated (hiPSC) mice were 570.5 ± 11.6 and 539.9 ± 21.9 mg/dL, respectively. 300 mg/dL is the threshold for murine diabetes.
**Figure S4.** hiPSC‐MSC increased hindlimb perfusion. TALLYHO hindlimb perfusion ratio (treated limb: untreated limb) increased more than control values out to 28 days post‐FAL. (*n* = 5/group).
**Figure S5.** Higher magnification (200X) representative images of PSR‐stained gastrocnemius muscle, as depicted in Figure 4, to show additional detail of stained collagen. This figure demonstrates the low background staining characteristic of all sections, as well as the high inter‐cellular PSR‐staining in more fibrosed tissues. Panel A = non‐ischemic control, Panel B = ischemic vehicle control, and Panel C = ischemic hiPSC‐MSC‐treated mouse.
**Figure S6.** Representative images (×200) of TALLYHO gastrocnemius CD206 positive cells at 30 days post‐injection from (A) vehicle treated, (B) hiPSC‐MSC treated (low density), and (C) hiPSC‐MSC treated (high‐density) tissue. Colorimetric detection was performed with DAB. Panel D shows gastrocnemius CD206‐positive cell density analysis results for vehicle (Veh) and hiPSC‐MSC (hiPSC) treated mice. Statistical comparison was made by Mann–Whitney Rank Sum *t*‐test.
**Table S1.** Summary of histological scoring for gastrocnemius muscle H&E sections.

## Data Availability

The data that support the findings of this study are available from the corresponding author upon reasonable request.
