# Supplementary material for: Human induced pluripotent stem cell‐derived mesenchymal stromal cells regenerate diabetic ischemic muscle
Source: Bioeng Transl Med. 2026 Feb 12;11(3):e70119. doi: 10.1002/btm2.70119 (PMC13247425; doi:10.1002/btm2.70119)
Supplement: Supplementary file 1 — Figure S1. Analysis of hiPSC‐MSC. (A, B) Morphology of hiPSC‐MSC. (C) Surface marker expression of hiPSC‐MSC as determined by flow cytometry. (D) Microscopic images of hiPSC‐MSC derived adiopocytes, osteoblasts, chondroblasts. Figure S2. Gel electrophoresis of PCR confirmation of mycoplasma‐free cell line. The left panel demonstrates no mycoplasma DNA in the hiPSC cell preparation prior to differentiation. The right panel demonstrates no mycoplasma DNA in the hiPSC cell preparation after MSC differentiation. Figure S3. hiPSC‐MSC administration did not affect serum glucose levels in diabetic phenotype. Average serum glucose concentrations, as determined via a glucometer, in the control and hiPSC‐MSC‐treated (hiPSC) mice were 570.5 ± 11.6 and 539.9 ± 21.9 mg/dL, respectively. 300 mg/dL is the threshold for murine diabetes. Figure S4. hiPSC‐MSC increased hindlimb perfusion. TALLYHO hindlimb perfusion ratio (treated limb: untreated limb) increased more than control values out to 28 days post‐FAL. (n = 5/group). Figure S5. Higher magnification (200X) representative images of PSR‐stained gastrocnemius muscle, as depicted in Figure 4, to show additional detail of stained collagen. This figure demonstrates the low background staining characteristic of all sections, as well as the high inter‐cellular PSR‐staining in more fibrosed tissues. Panel A = non‐ischemic control, Panel B = ischemic vehicle control, and Panel C = ischemic hiPSC‐MSC‐treated mouse. Figure S6. Representative images (×200) of TALLYHO gastrocnemius CD206 positive cells at 30 days post‐injection from (A) vehicle treated, (B) hiPSC‐MSC treated (low density), and (C) hiPSC‐MSC treated (high‐density) tissue. Colorimetric detection was performed with DAB. Panel D shows gastrocnemius CD206‐positive cell density analysis results for vehicle (Veh) and hiPSC‐MSC (hiPSC) treated mice. Statistical comparison was made by Mann–Whitney Rank Sum t‐test. Table S1. Summary of histological scoring for gastrocnemius muscle H& [file BTM2-11-e70119-s001.docx]

**SUPPLEMENTAL MATERIAL**

**Human Induced Pluripotent Stem Cell-Derived Mesenchymal Stromal Stem Cells Regenerate Diabetic Ischemic Muscle**

**Short title: hiPSC-MSC Regenerate Diabetic Ischemic Muscle**

**
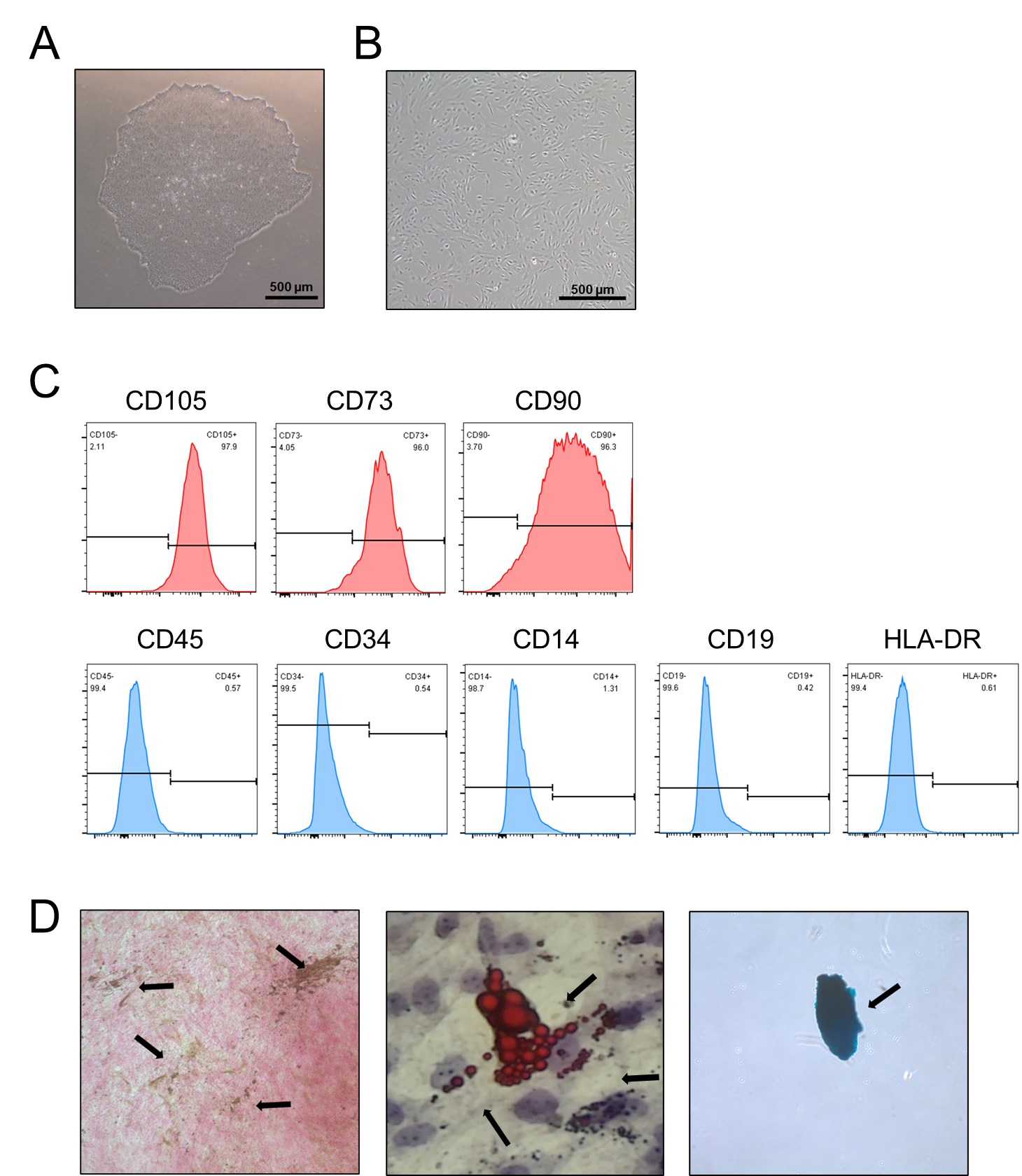
**

**Supplemental Figure 1.** Analysis of hiPSC-MSC. (A, B) Morphology of hiPSC-MSC. (C) Surface marker expression of hiPSC-MSC as determined by flow cytometry. (D) Microscopic images of hiPSC-MSC derived adiopocytes, osteoblasts, chondroblasts.


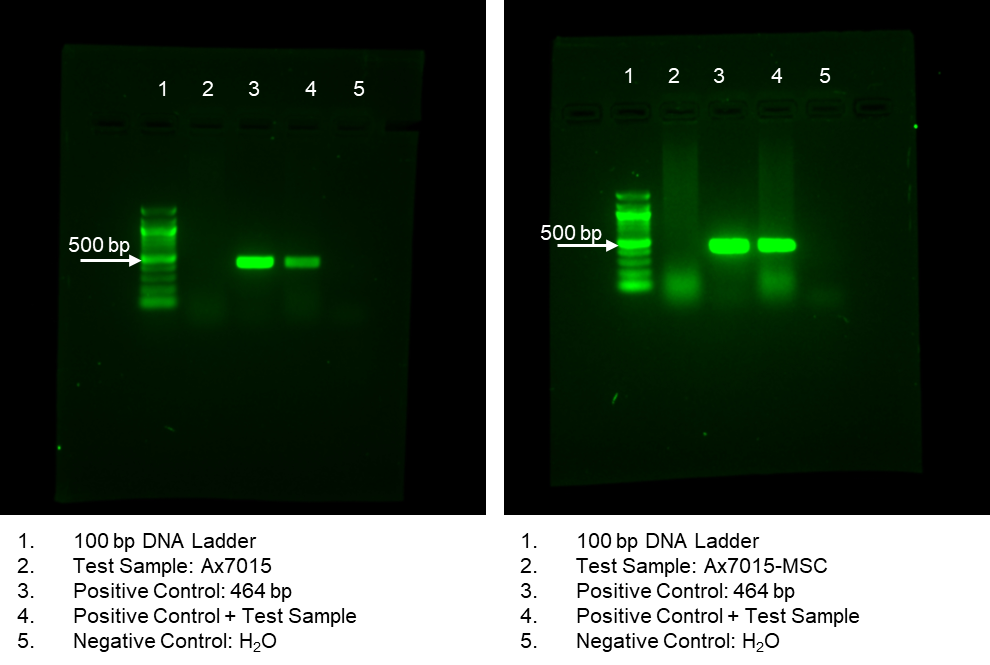


**Supplemental Figure 2.** Gel electrophoresis of PCR confirmation of mycoplasma-free cell line. The left panel demonstrates no mycoplasma DNA in the hiPSC cell preparation prior to differentiation. The right panel demonstrates no mycoplasma DNA in the hiPSC cell preparation after MSC differentiation.

**
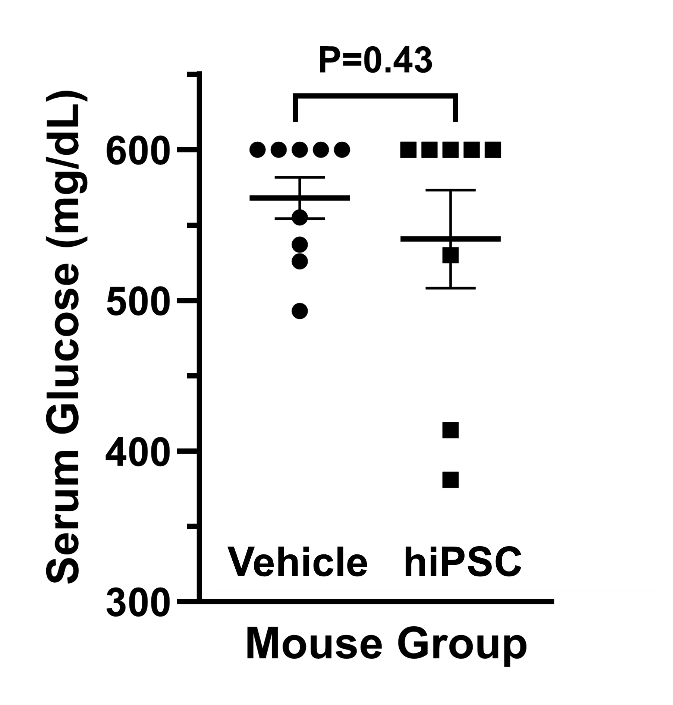
**

**Supplemental Figure 3.** hiPSC-MSC administration did not affect serum glucose levels in diabetic phenotype. Average serum glucose concentrations, as determined via a glucometer, in the control and hiPSC-MSC-treated (hiPSC) mice were 570.5±11.6 and 539.9±21.9 mg/dL, respectively. 300 mg/dL is the threshold for murine diabetes.

**
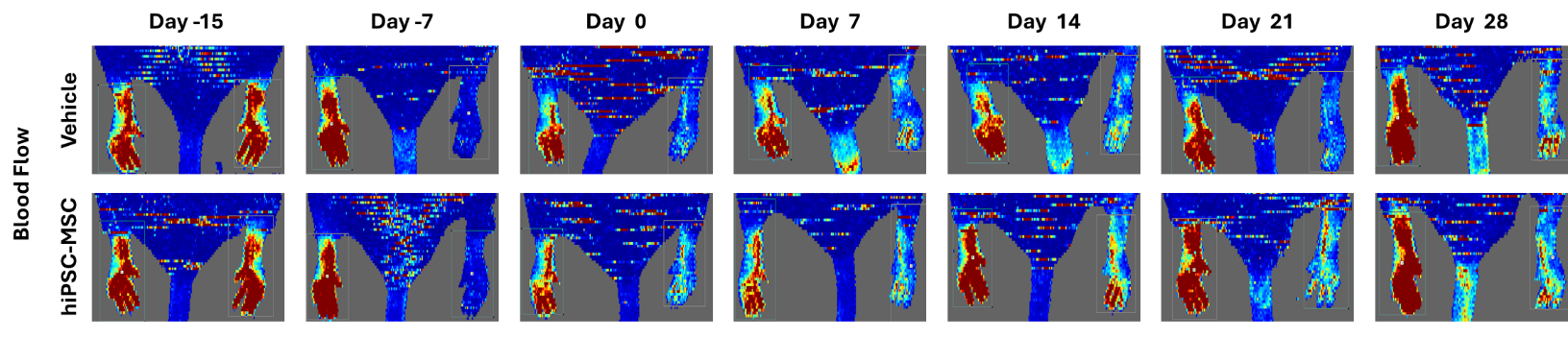
**

**Supplemental Figure 4.** hiPSC-MSC increased hindlimb perfusion. TALLYHO hindlimb perfusion ratio (treated limb:untreated limb) increased more than control values out to 28 days post-FAL. (n=5/group).


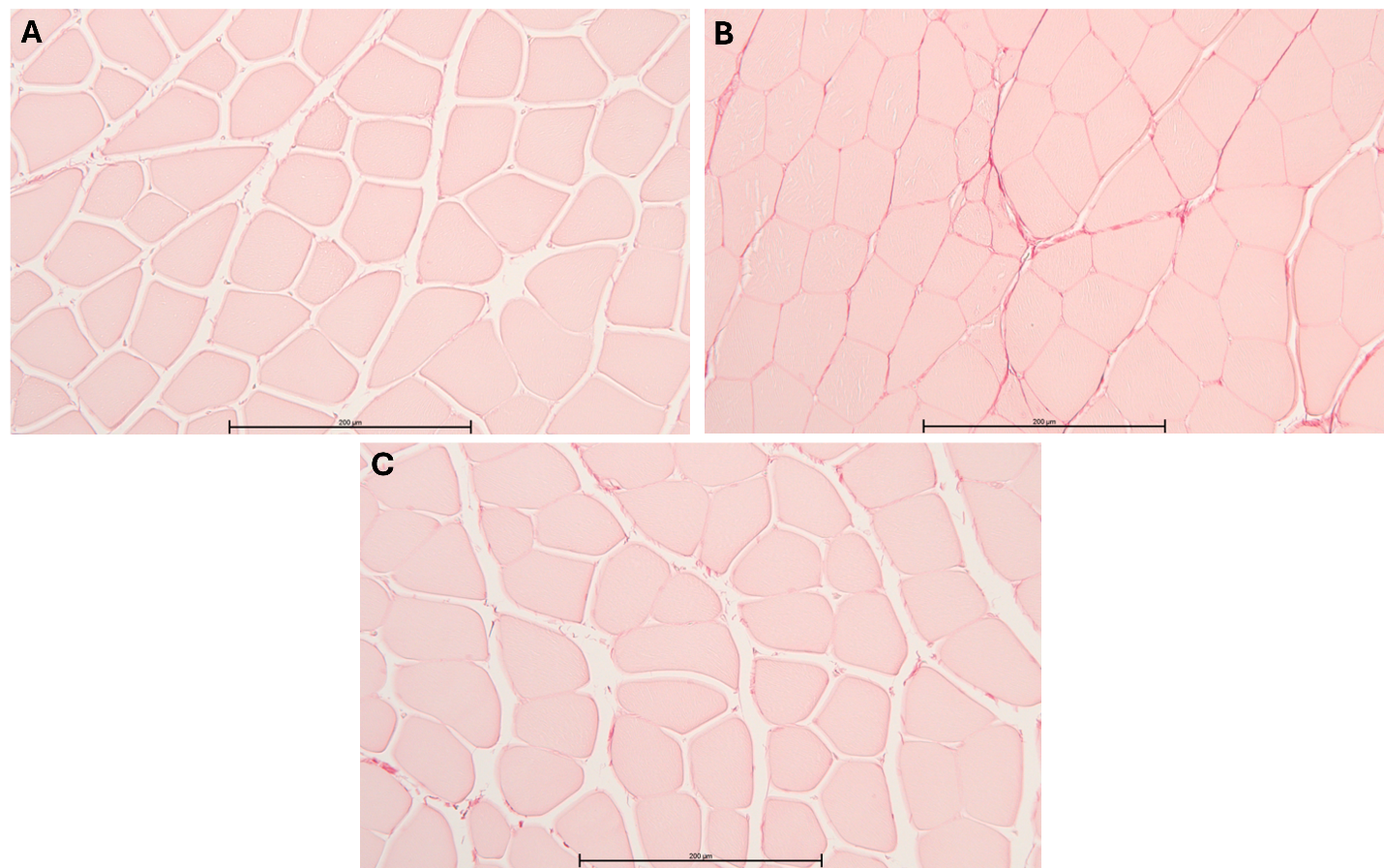


**Supplemental Figure 5.** Higher magnification (200X) representative images of PSR-stained gastrocnemius muscle, as depicted in Figure 4, to show additional detail of stained collagen. This figure demonstrates the low background staining characteristic of all sections, as well as the high inter-cellular PSR-staining in more fibrosed tissues. Panel A=non-ischemic control, Panel B=ischemic vehicle control, and Panel C=ischemic hiPSC-MSC-treated mouse.


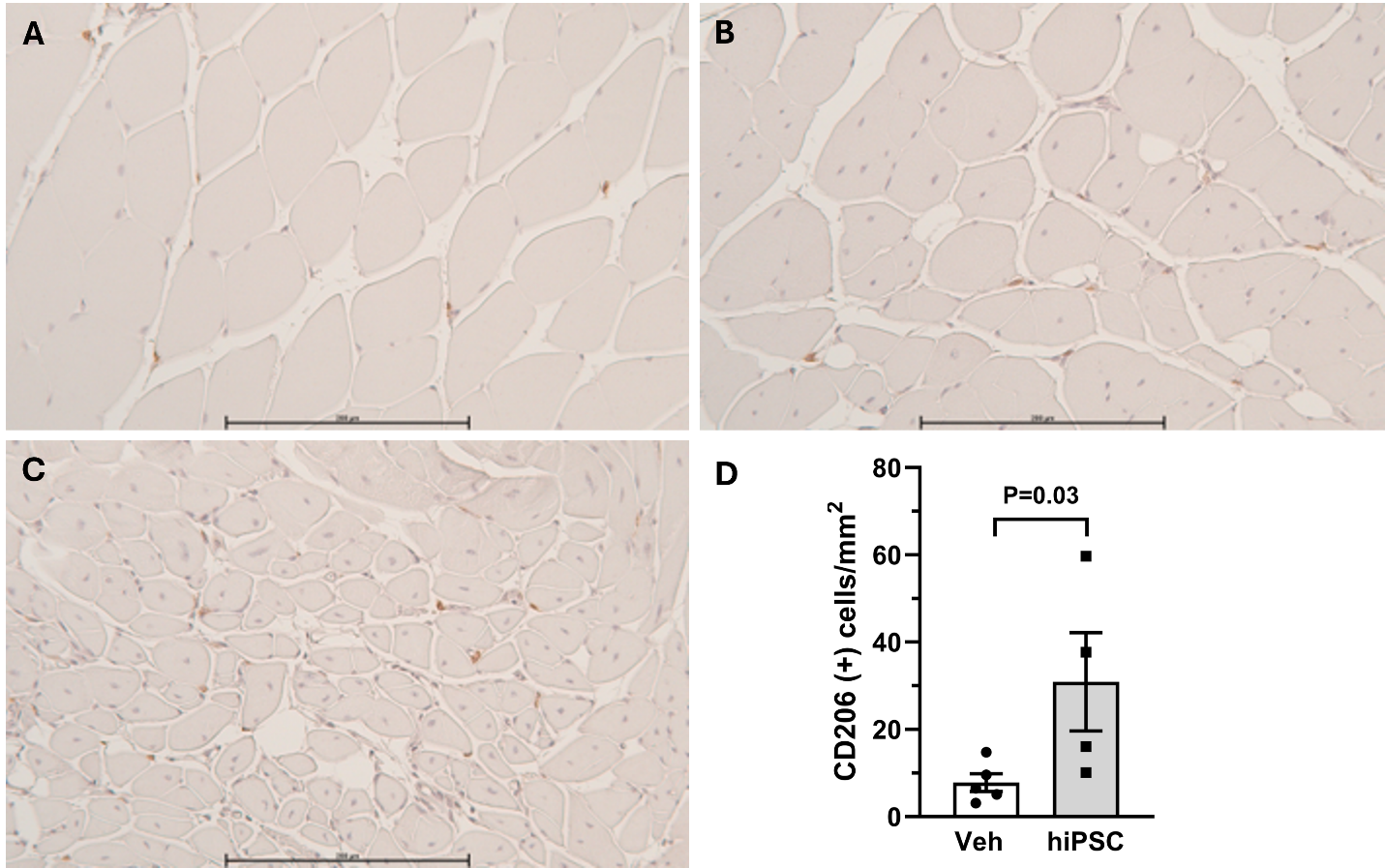


**Supplemental Figure 6.** Representative images (200X) of TALLYHO gastrocnemius CD206 positive cells at 30 days post-injection from A) vehicle treated, B) hiPSC-MSC treated (low density) and C) hiPSC-MSC treated (high-density) tissue. Colorimetric detection was performed with DAB. Panel D shows gastrocnemius CD206-positive cell density analysis results for vehicle (Veh) and hiPSC-MSC (hiPSC) treated mice. Statistical comparison was made by Mann-Whitney Rank Sum t-test.

**Supplemental Table 1.** Summary of histological scoring for gastrocnemius muscle H&E sections.

| **Pathology** | **Control** | **Vehicle** | **hiPSC-MSC** |
| --- | --- | --- | --- |
| Fiber Size Variation | 0.46±0.18 | 2.54±0.35* | 2.25±0.31* |
| Nuclear Centralization | 0.54±0.18 | 2.54±0.33* | 2.13±0.30* |
| Fatty Deposition | 0±0 | 1.31±0.26* | 0.5±0.38^+^ |
| Cellular Infiltrates | 0.08±0.08 | 1.54±0.37* | 1.13±0.30 |
| Enlarged Nuclei | 0.54±0.18 | 2.54±0.33* | 2.13±0.30* |

N=13, 13, and 8 for control, vehicle, and hiPSC-MSC-treated mouse mean values, respectively. *P<0.05 vs. Control (One-way ANOVA), ^+^P=0.03 vs. Vehicle (Mann-Whitney Rank Sum Test).
